# Gilteritinib Enhances Anti-Tumor Efficacy of CDK4/6 Inhibitor, Abemaciclib in Lung Cancer Cells

**DOI:** 10.3389/fphar.2022.829759

**Published:** 2022-06-23

**Authors:** Chao-Yue Sun, Milton Talukder, Di Cao, Cun-Wu Chen

**Affiliations:** ^1^ College of Biological and Pharmaceutical Engineering, West Anhui University, Lu’an, China; ^2^ Department of Physiology and Pharmacology, Faculty of Animal Science and Veterinary Medicine, Patuakhali Science and Technology University, Barishal, Bangladesh; ^3^ State Key Laboratory of Oncology in South China, Department of Radiology, Sun Yat-Sen University Cancer Center, Key Laboratory of Nasopharyngeal Carcinoma Diagnosis and Therapy, Guangzhou, China

**Keywords:** lung cancer, CDK4/6, abemaciclib, gilteritinib, RB, Akt

## Abstract

Abemaciclib is a cyclin-dependent kinases 4/6 (CDK4/6) inhibitor approved for the treatment of metastatic breast cancer. Preclinical studies suggest that abemaciclib has the potential for lung cancer treatment. However, several clinical trials demonstrate that monotherapy with abemaciclib has no obvious superiority than erlotinib to treat lung cancer patients, limiting its therapeutic options for lung cancer treatment. Here, we show that the US Food and Drug Administration (FDA)-approved drug, gilteritinib, enhances the cytotoxicity of abemaciclib through inducing apoptosis and senescence in lung cancer cells. Interestingly, abemaciclib in combination with gilteritinib leads to excessive accumulation of vacuoles in lung cancer cells. Mechanistically, combined abemaciclib and gilteritinib induces complete inactivation of AKT and retinoblastoma (Rb) pathways in lung cancer cells. In addition, RNA-sequencing data demonstrate that combination of abemaciclib and gilteritinib treatment induces G2 phase cell-cycle arrest, inhibits DNA replication, and leads to reduction in homologous recombination associated gene expressions. Of note, abemaciclib-resistant lung cancer cells are more sensitive to gilteritinib treatment. In a mouse xenograft model, combined abemaciclib and gilteritinib is more effective than either drug alone in suppressing tumor growth and appears to be well tolerated. Together, our findings support the combination of abemaciclib with gilteritinib as an effective strategy for the treatment of lung cancer, suggesting further evaluation of their efficacy is needed in a clinical trial.

## Introduction

Lung cancer remains the most common cause of cancer-related deaths worldwide, with an estimated more than 1.8 million deaths in 2020 (Sung et al., 2021). Despite an application of standard therapies targeting EGFR, ALK, BRAF, HER2, RET, ROS1, and MET, only less than 25% of lung cancer patients experience life-altering survival from these therapies, and drug resistance inevitably develops during treatments (Wang et al., 2021). Dysregulated cell division facilitates uncontrolled proliferation that is a typical hallmark of cancer, and targeting cell cycle represents an effective therapeutic strategy for cancer (O'Leary et al., 2016). Cyclin dependent kinases 4/6 (CDK4/6) are the most important cycle players that commit cell to divide by specifically promoting G1 to S-phase cell cycle transition through phosphorylation of retinoblastoma (Rb) (Bertoli et al., 2013). Of note, dysregulations of cell cycle are detected in over 90% of lung cancers (Zhang et al., 2021), and aberrant alteration of CDK4/6 pathways are detected in all lung cancer patients (Gopalan et al., 2018). Currently, FDA-approved CDK4/6 inhibitors, including ribociclib, abemaciclib and palbociclib, have significant therapeutic activity against lung cancer cells in the preclinical studies (Schettini et al., 2018; Hino et al., 2020; La Monica et al., 2020).

In a clinical trial, patients with KRAS-mutant lung cancer displayed response to abemaciclib, with a safety profile (Patnaik et al., 2016). In a recent randomized phase- III study, however, lung cancer patients with KRAS mutation received either erlotinib or abemaciclib monotherapy. Despite a significant improved progression-free survival (PFS), abemaciclib showed no better overall survival (OS) compared to erlotinib that is no longer used for the treatment of lung cancer due to its poor efficacy (Goldman et al., 2020). In another phase-Ⅱ trial, advanced lung cancer patients showed no obvious response to palbociclib, and only patients with p16-null were eligible (Gopalan et al., 2018). Thus, these clinical trials suggest that monotherapies targeting CDK4/6 are not very effective treatment for lung cancer patients, and CDK4/6 inhibitors combining with other drugs rank as a reasonable strategy. Of note, the use of CDK4/6 inhibitors in combination with mitogen-activated extracellular signal-regulated kinase (MEK), or mammalian target of rapamycin (mTOR) inhibitors for treating lung cancer are being tested in clinical trials (NCT03170206, NCT02857270).

Gilteritinib is an orally available FDA-approved drug used for the treatment of patients with acute myeloid leukaemia (AML) harboring FMS-like tyrosine kinase 3 (FLT3) mutation (Dhillon, 2019; Levis and Perl, 2020). Approximately 28% of AML patients have constitutively FLT3 mutation, and these patients displayed higher rates of relapse, and poorer prognosis (Ley et al., 2013; Daver et al., 2021). Gilteritinib is a selective second-generation FLT3 inhibitor, with a high overall response rate (ORR, 52%) in FLT3^mut+^ AML patients, and more than 90% of FLT3 signaling inhibition was observed in most patients receiving a daily dose of 80 mg gilteritinib (Perl et al., 2017). In addition to FLT3, gilteritinib also inhibits another receptor tyrosine kinase (RTK), AXL that is frequently over-expressed in AML (Lee et al., 2017). Interestingly, AXL facilitates FLT3 activation in AML, which is involved in drug resistance of FLT3 inhibitors (Gorcea et al., 2018). Thus, gilteritinib is a dual FLT3 and AXL inhibitor. Moreover, preclinical studies demonstrate that gilteritinib exhibits inhibitory activity against c-Kit, AKT, and sodium-coupled neutral amino acid transporter 1 (SNAT1) pathways in AML cells (Mori et al., 2017; Zavorka Thomas et al., 2021).

In the present study, we investigated the antitumor effect of abemaciclib alone, or in combination with gilteritinib in lung cancer cells, and demonstrated that gilteritinib enhances the cytotoxicity of abemaciclib *in vitro* and *in vivo*. Our findings support the use of abemaciclib in combination with gilteritinib for the treatment of lung cancer.

## Materials and Methods

### Reagents and Antibodies

Gilteritinib (T4409), quizartinib (T2066) and OSI-027 (T6319) were purchased from TargetMol. Palbociclib (S1579) and rapamycin (S1039) were purchased from Selleck, abemaciclib (L190223) and Torin 1 (T129642) were obtained from Aladdin. Primary antibodies for Western blot analysis include AKT (CST, 4691S), phospho-AKT (ser47, CST, 4060S), CDK2 (CST, 2546S), Rb (Abcam, ab181616), phospho-Rb (Ser807/811, CST, 8516T), phospho-Rb (Ser780, CST, 8180T), Cyclin D1 (Abcam, ab40754), GAPDH (CST, 5174S). The anti-rabbit HRP-conjugated secondary antibodies (7074S) were obtained from CST.

### Cell Lines and Cell Culture

Human lung cancer cell lines, including A549, H1650 and PC9, and colorectal cancer cells, HCT115, and esophageal cancer cells, KYSE30, were obtained from State Key Laboratory of Oncology in South China. All cell lines were cultured in RPMI-1640 medium (Gibco, United States), supplemented with 10% FBS (Gibco, United States), and maintained at 37°C in a standard incubator (5% CO_2_). In addition, abemaciclib-resistant A549 cells were developed through serial passaging with dose-escalation of abemaciclib for 3 months by continuous drug exposure (Pesch et al., 2020; Sun et al., 2021b).

### Cell Viability

Cell viability was measured as described previously (Sun et al., 2021b). Briefly, cells were seeded into 96-well plates at 4,000 cells per well, and continuously cultured for 24 h. Then, cells were treated with abemaciclib or gilteritinib alone, or in combination. After treatment, 10 μL Cell Counting Kit-8 (CCK-8, APExBIO) solution was added to each well and cells were incubated for 3 h. The optical absorbance was measured at 450 nm using a microplate reader.

### EdU Assay

Cells were seeded into six-well plates at 2 × 10^5^ cells per well, and then treated with abemaciclib or gilteritinib alone, or in combination for 48 h. After treatment, cells were incubated with 10 μM EdU (Beyotime, C0071S) for 3 h. Cells were washed with PBS for three times, and then fixed in 4% paraformaldehyde for 15 min. After PBS washing, cells were incubated with 0.5% Triton X-100 at room temperature for 10 min. 500 μL click reaction buffer was added to each well, and cells were incubated in the dark for 30 min. Then, cells were stained with DAPI, and EDU-positive cells were visualized by a fluorescence microscope.

### Senescence Assay

Cell senescence was determined using a SA-β-Gal staining kit (Beyotime, C0602) according to the manufacturer’s protocol. Briefly, cells were plated in six-well plates at 2 × 10^5^ cells per well and treated with abemaciclib alone, or in combination with gilteritinib for 48 h. After washing with PBS, cells were fixed at room temperature for 15 min. After washing, 1 ml SA-β gal solution was added to each well, and cells were incubated at 37°C overnight. Cells were photographed using an electron microscope.

### Apoptosis Assay

Apoptosis was performed using Annexin V-AF647/PI staining kit (ESscience, AP006) according to the manufacturer’s protocol. Briefly, cells were seeded in six-well plates at 2 × 10^5^ cells per well and treated with abemaciclib alone, or in combination with gilteritinib for 72 h. After washing with PBS, cells were stained with 5 μL Annexin V-FITC and 10 μL PI in the dark condition for 5 min. Then, apoptosis was measured using a Flow Cytometer (BD).

### Cell-Cycle Analysis

Cell cycle staining kit (MultiSciences, 70-CCS012) was employed to detect cell cycle. Briefly, cells were seeded in six-well plates at 2 × 10^5^ cells per well and treated with abemaciclib alone, or in combination with gilteritinib for 24 h. After treatment, cells were centrifuged, and suspended in 1 ml DNA staining solution. Cells were stained with 10 μL permeabilization solution in the dark room for 30 min. Cell Samples were analysed by the ACEA NovoCyte Flow Cytometer (Agilent).

### Western Blot

Western blot analysis was performed as previously described (Sun et al., 2019; Sun et al., 2021a). Briefly, cells were seeded into the six-well plates and then treated with abemaciclib alone, or in combination with gilteritinib for 48 h. Following treatment, cells were washed with PBS, followed by lysis in RIPA buffer (containing protease inhibitors and phosphatase inhibitors). Protein concentration was determined by a BCA protein assay kit (Thermo Fischer). Equal amount of protein (20 μg) was subjected to SDS-PAGE electrophoresis, and then transferred onto 0.45 mm PVDF membranes (Millipore, United States). After transfer, membranes were blocked with skim milk for 1 h at room temperature, and incubated with primary antibodies overnight at 4°C in refrigerator. After washing with TBST, blots were incubated with secondary antibodies for 1 h. Bands were visualized using an enhanced chemiluminescence kit (Thermo Fischer) in Bio-Rad ChemiDoc Imaging System.

### RNA Extraction and Real-Time qPCR

Cells were treated with abemaciclib alone, or in combination with gilteritinib for 48 h. Total RNA was extracted using TRIzol reagent (Invitrogen, 15596018), according to the manufacturer’s instruction. Then, cDNA was obtained using the GoScript Reverse Transcriptase kit (Promega, A5004). Real-time quantitative PCR (qRT-PCR) analysis was performed with GoTaq qPCR Master Mix kit (Promega, A6002) using Real-Time PCR System. Relative expression was calculated by the 2^−ΔΔCt^ method. The primers used in this study are shown in Table 1.

**TABLE 1:** The primer sequences of DNA replication and HR genes used for qRT-PCR.

Gene	Forward	Reverse
MCM4	GAG​TAA​TGT​GAA​GTC​TGT​CT	GAAGCAAGCCTCTCATAA
MCM6	CAA​TGG​CTA​CAA​TGA​AGA​CA	GGT​TAG​AGA​TTC​GGC​AGT​A
MCM7	ACT​ACA​TCA​CAG​CAG​CAT​A	CCACATCCACCATTCTCA
POLD3	CTCCTGCTGAATCCTCTT	CATTGTTGGCTTGTGTCT
RFC4	TTC​AAC​AGC​AGC​GAT​TAC​TA	GTA​GCG​CTT​TGA​AGA​AAT​GT
PCNA	CTG​AGG​GCT​TCG​ACA​CCT​AC	CTT​CGG​CCC​TTA​GTG​TAA​TG
BRCA1	AAG​GCA​AGA​TCT​AGA​GGG​A	GGT​TGA​AGA​TGG​TAT​GTT​G
BRCA2	ATA​CAG​TTA​GCA​GCG​ACA​A	TCC​ACC​TCA​GAA​CAA​GAT​G
RAD51	AGTGGCTGAGAGGTATGG	TCTGGTGGTCTGTGTTGA
RAD54L	TCC​TGT​GAT​GAT​GAA​GAC​T	GGTGGTTGATTGGTTAGC
BRIP1	AGA​GGA​GGC​AAG​AGA​AGT​A	AGG​AGT​AAG​TCT​GTT​GAA​TCG
BARD1	TCA​ATA​CAG​TCG​CAT​ACC​A	TGTCAAGAGGAAGCAACT
XRCC2	TGTGTAGTGCCTTCCATA	TCGTGCTGTTAGGTGATA
RBBP8	TTG​ATC​GGA​CAA​CAC​ATG​AA	CCA​TGA​GTG​TGT​AGT​TTC​TT

### RNA Sequencing and Analysis

A549 cells were treated with abemaciclib alone, or in combination with gilteritinib for 48 h. After treatment, total RNA was isolated using TRIzol reagent. RNA libraries and sequencing were performed by the Novogene Bioinformatics Technology (Beijing) using an Illumina HiSeq2500. Following trimming and filtration, sequence data were aligned with STAR aligner and counted with HTSeq. DESeq2 was used to determine the differential expressions of genes. DESeq2 results were analyzed using a *p* < 0.05 significance followed by performance of false discovery rates analysis. Differentially expressed genes were subjected to the Kyoto Encyclopedia of Genes and Genomes (KEGG), disease ontology (DO) and Reactome analysis.

### *In Vivo* Xenograft Model

All procedures involving animal experiment were strictly approved by the Animal Institutional Care and Use Committee (IACUC) of Sun Yat-Sen University (ethical ID: L102012020120J). Female BALB/c nude mice (4–6 weeks, 18–22 g) were provided by Guangdong medical laboratory animal center (Foshan, China). A549 cells (4 × 10^6^) were subcutaneously injected into nude mice. When tumors volume reached 100 mm^3^, mice were randomly divided into four groups (*n* = 10): control, abemaciclib (40 mg/kg), gilteritinib (2.5 mg/kg), abemaciclib + gilteritinib. Mice in treatment groups were administered daily *via* oral gavage with abemaciclib alone, or gilteritinib alone, or their combination; while mice in control group were treated with the vehicle for continuous 20 days. Abemaciclib and gilteritinib were first dissolved in DMSO and then diluted in water with 0.4% carboxymethylcellulose (Merck millipore) and 0.2% Tween-80 (Sigma). During drug treatment, tumor volume (Length × width^2^ × 0.5) was measured every 2 days, and tumors were collected after treatment.

### Statistical Analysis

All data are presented as means ± standard deviation (SD). GraphPad Prism software (version 8.0) was used for statistical analysis. To compare two groups, Student’s t-test was used, and one-way analysis of variance (ANOVA) was used for multiple group comparisons. *p* < 0.05 was considered significant.

## Results

### Gilteritinib Sensitizes Lung Cancer Cells to CDK4/6 Inhibitor, Abemaciclib, but Not Palbociclib

Since CDK4/6 inhibitors inevitably develop drug resistance (Pandey et al., 2019), and monotherapy with CDK4/6 inhibitors, including abemaciclib is not very effective for the treatment of lung cancer patients (Gopalan et al., 2018), drug combination is thus a reasonable therapeutic strategy. To identify potential drugs that might sensitize abemaciclib, we employed FDA-approved small-molecule protein kinase inhibitors to co-treat with abemaciclib in lung cancer cells (Roskoski, 2020), and found that mTOR inhibitor, rapamycin and FLT3 inhibitor, gilteritinib enhanced the cytotoxicity of abemaciclib (Supplementary Figure S3C). Because mTOR inhibitors in combination with CDK4/6 inhibitors was reported (Olmez et al., 2017), we thus investigated the effect of the combination of abemaciclib and gilteritinib in lung cancer cells.

**FIGURE 1:**
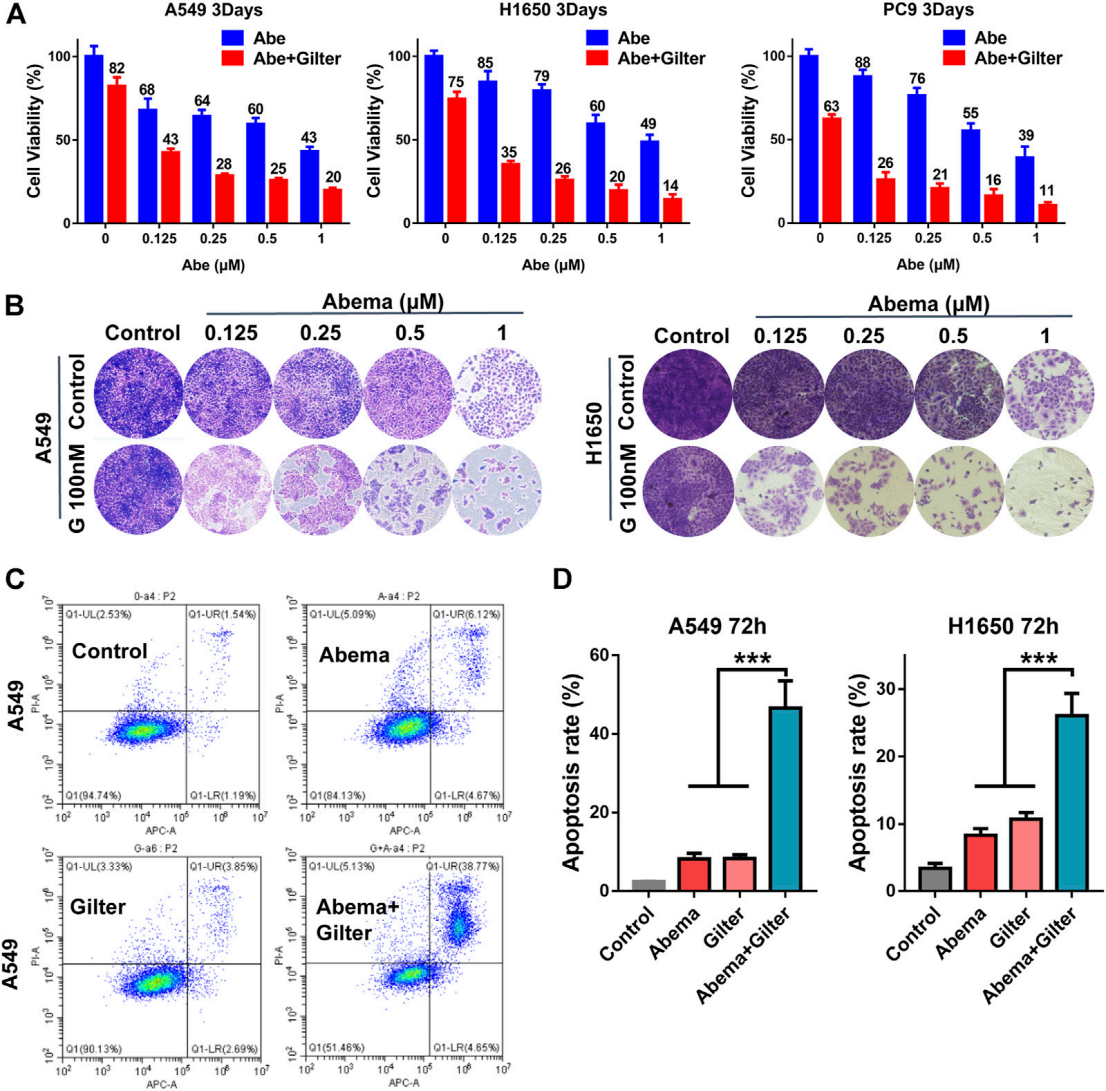
Gilteritinib sensitizes lung cancer cells to CDK4/6 inhibitor, abmaciclib **(A)** A549, H1650, PC9 cells were treated by increasing concentrations of abemaciclib with or without gilteritinib for 72 h, and the cell viability was measured using CCK-8 assay **(B)** A549, H1650 cells were treated by abemaciclib with or without gilteritinib for 72 h, and cell proliferation was measured using crystal violet staining **(C,D)** A549, H1650 cells were treated by 1 μM abemaciclib, or 150 nM gilteritinib alone, or their combination, and apoptosis was detected by flow cytometer. Data are representative of at least three independent experiments. ***p < 0.0001.

**FIGURE 2:**
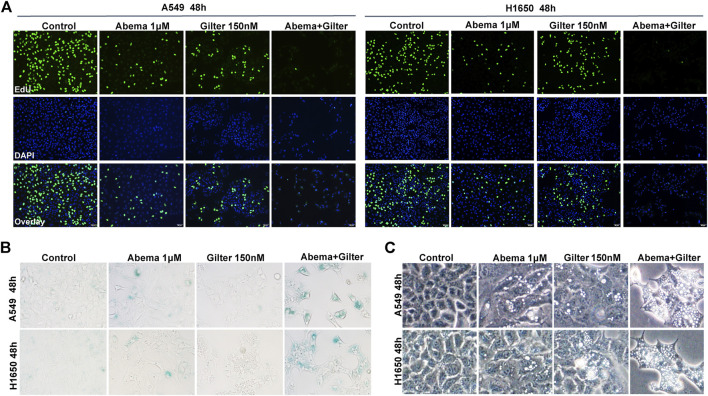
Abemaciclib in combination with gilteritinib inhibits cell proliferation, induces senescence and vacuoles in lung cancer cells **(A)** A549, H1650 cells were treated by 1 μM abemaciclib, or 150 nM gilteritinib, or in combination, and cell proliferation was determined by the EdU assay **(B)** A549, H1650 cells were treated by 1 μM abemaciclib, or 150 nM gilteritinib, or their combination, and the SA-β gal staining kit was used to detect cell senescence **(C)** A549 and H1650 cells were treated by 1 μM abemaciclib, or 150 nM gilteritinib alone, or the combination, and cellular morphology was observed using microscope. Data are representative of at least three independent experiments.

CCK8 assay data showed that low concentration of gilteritinib (0.1 μM) combined with abemaciclib suppressed proliferation in A549, H1650, PC9 cells (Figure 1A). In line with this, crystal violet staining results demonstrated that lung cancer cells displayed more susceptible to abemaciclib treatment, in the presence of gilteritinib (Figure 1B). In addition, EdU assay showed that abemaciclib or gilteritinib alone reduced the rate of EdU-positive cells in both A549 and H1650 cells (Figure 2A). Notably, EdU failed to insert cells in response to the combined treatment (Figure 2A), suggesting that combined treatment robustly inhibited proliferation in lung cancer cells. Moreover, abemaciclib or gilteritinib alone slightly induced apoptosis in A549 and H1650 cells, and apoptosis rate was significantly enhanced by the combined treatment, compared with either drug (Figures 1C,D). We then explored if the combined treatment was equally applicable to other cancer types, and found that abemaciclib in combination with gilteritinib had stronger cytotoxicity than single drug treatment in colorectal cancer cells, HCT115, and esophageal squamous cell carcinoma, KYSE30 (Supplementary Figure S1A). Besides, we interrogated whether the synergy occurs between gilteritinib and another CDK4/6 inhibitor, palbociclib. Unlike abemaciclib, palbociclib failed to sensitize gilteritinib in lung cancer cells (Supplementary Figure S1B). In similar with gilteritinib, quizartinib improved the anti-cancer effect of abemaciclib in lung cancer cells (Supplementary Figure S1C), indicating that FLT3 might confer synthetic lethality to CDK4/6 inhibition. We generated abemaciclib-resistant A549 cells and found that abemaciclib-resistant A549 cells were sensitive to gilteritinib treatment (Supplementary Figure S1F). Taken together, abemaciclib in combination with gilteritinib inhibits cell proliferation and induces apoptosis in lung cancer cells.

### Abemaciclib in Combination With Gilteritinib Induces Senescence and Cytoplasmic Vacuoles in Lung Cancer Cells

Although cellular senescence is a double-edged sword for cancer treatment, induction of senescence is considered as a therapeutic opportunity for cancer by triggering the senescence-associated secretory phenotype (SASP) (Faget et al., 2019; Wang et al., 2019). Since Rb pathway plays a critical role in senescence, CDK4/6 inhibitors have ability to induce senescence in cancer cells (Wagner and Gil, 2020). To determine whether gilteritinib enhances abemaciclib-induced cell senescence in lung cancer cells, we detected the SA-β-gal, an important marker of senescence. Compared to control cells, abemaciclib alone indeed induced a slight increase of SA-β-gal activity, while gilteritinib treatment showed no effect (Figure 2B). Of note, abemaciclib in combination with gilteritinib accelerated cell senescence, compared to either drug alone treatment (Figure 2B).

It has been observed that abemaciclib induced cell death by inducing cytoplasmic vacuoles in cancer cells, and massive vacuolization led to atypical cell death, called methuosis (Huang et al., 2018; Hino et al., 2020; Liang et al., 2020). Here, we found that abemaciclib treatment caused a striking accumulation of cytoplasmic vacuoles in A549 and H1650 cells, when compared with untreated cells (Figure 2C). Interestingly, gilteritinib treatment showed similar activity with abemaciclib to induce cytoplasmic vacuoles in lung cancer cells (Figure 2C). Importantly, abemaciclib in combination with gilteritinib induced more vacuoles than either drug (Figure 2C). In addition, similar data were observed in other cancer cell lines, including colorectal cancer cell line, HCT15 (Supplementary Figure S1D). As described above, another CDK4/6 inhibitor, palbociclib exhibited no synergism with gilteritinib in lung cancer cells (Supplementary Figure S1B). Interestingly, we found that palbociclib alone treatment failed to induce cytoplasmic vacuoles, and palbociclib inhibited gilteritinib-induced vacuoles in lung cancer cells (Supplementary Figure S1F), suggesting that excessive vacuoles might trigger cell death. Thus, combined abemaciclib and gilteritinib treatment induces senescence and cytoplasmic vacuoles in lung cancer cells.

### Abemaciclib in Combination With Gilteritinib Inhibits Rb and AKT Pathways

**FIGURE 3:**
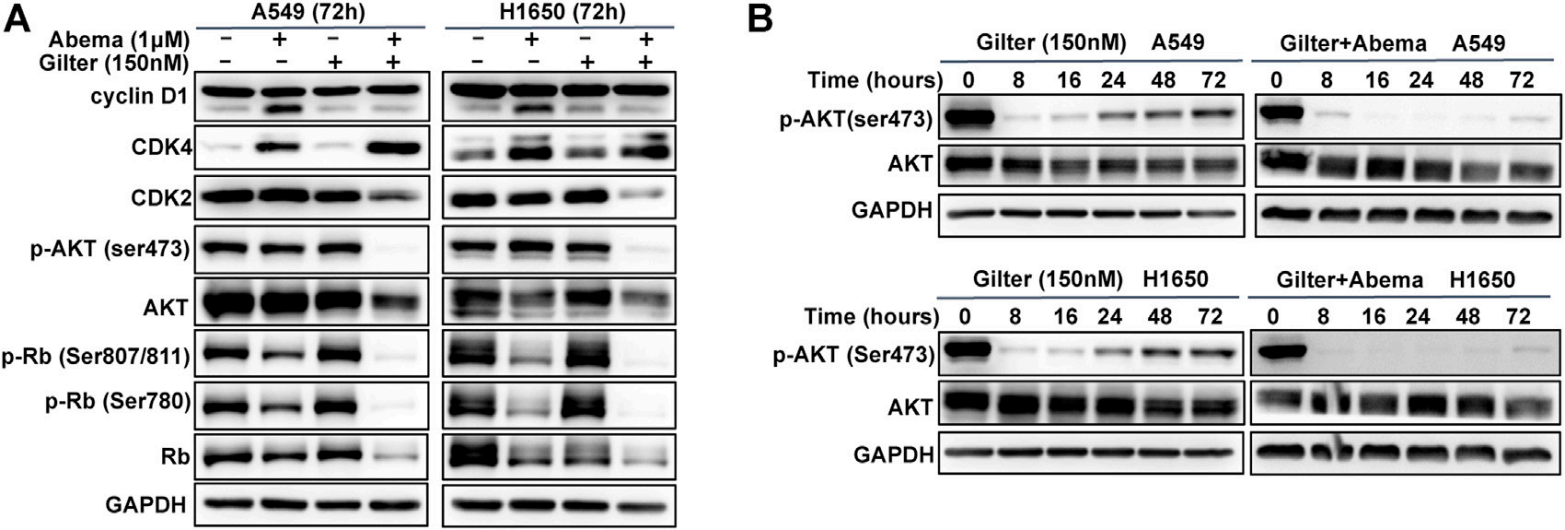
Abemaciclib in combination with gilteritinib inhibits Rb and AKT pathways **(A)** A549, H1650 cells were treated by 1 μM abemaciclib, or 150 nM gilteritinib alone, or their combination for 72 h, and Western blot was used to determine the expressions of cyclin D1, CDK2, CDK4, p-AKT (ser473), AKT, p-Rb (ser807/811, ser780) and Rb **(B)** A549 and H1650 cells were treated by gilteritinib (150 nM) alone, or combined with abemaciclib for increasing time points, and Western blot was used to determine the expressions of p-AKT (ser473) and AKT, GAPDH was used as a loading control.

It is clear that CDK4/6 pathway mediates cycle transition from G1 to S-phase through phosphorylation of Rb (O'Leary et al., 2016). To investigate the molecular mechanism of the anti-cancer activity of combined abemaciclib and gilteritinib, we used Western blot analysis to detect Rb pathway. As shown in Figure 3A, as expected, abemaciclib alone treatment reduced phospho-Rb at ser807/811 and ser780 sites, but phospho-Rb remained unchanged in response to gilteritinib alone treatment. Notably, the combined treatment almost completely inhibited phospho-Rb at both sites and total Rb in A549 and H1650 cells (Figure 3A, and Supplementary Figure S3A), suggesting that combination treatment might promote Rb proteasomal degradation, thereby resulting in inhibition of p-Rb. In addition, as previously reported (Chen et al., 2018), abemaciclib increased the expression of cyclin D1, however, this was completely prevented by the addition of gilteritinib (Figure 3A). Surprisingly, abemaciclib alone or combined with gilteritinib led to a significant increase of CDK4 expression (Figure 3A), which was consistent with previous report (Naz et al., 2018). However, the combined treatment with abemaciclib and gilteritinib effectively reduced the expression of CDK2 in A549 and H1650 cells (Figure 3A). Surprisingly, after 72 h treatment, combined treatment caused a complete inhibition of AKT phosphorylation in lung cancer cells, whereas treatment with either abemaciclib or gilteritinib showed no obvious effect on AKT pathway (Figure 3A). However, previous studies have demonstrated that gilteritinib inhibited AKT pathway in cancer cells (Li et al., 2020). Thus, we examined the effect of gilteritinib on phosphorylation of AKT in various time points using Western blot. As shown in Figure 3B, after 8 h treatment with gilteritinib alone, AKT is inactivated in A549 and H1650 cells, but AKT reactivation was observed after 24 h treatment. Of note, the addition of abemaciclib completely resisted the AKT reactivation (Figure 3B). Taken together, these results indicate that combined abemaciclib and gilteritinib treatment inhibited Rb and AKT pathways in lung cancer cells.

### Abemaciclib in Combination With Gilteritinib Induces Cell Cycle Arrest at G2 Phase and Inhibits DNA Replication, Homologous Recombination

**FIGURE 4:**
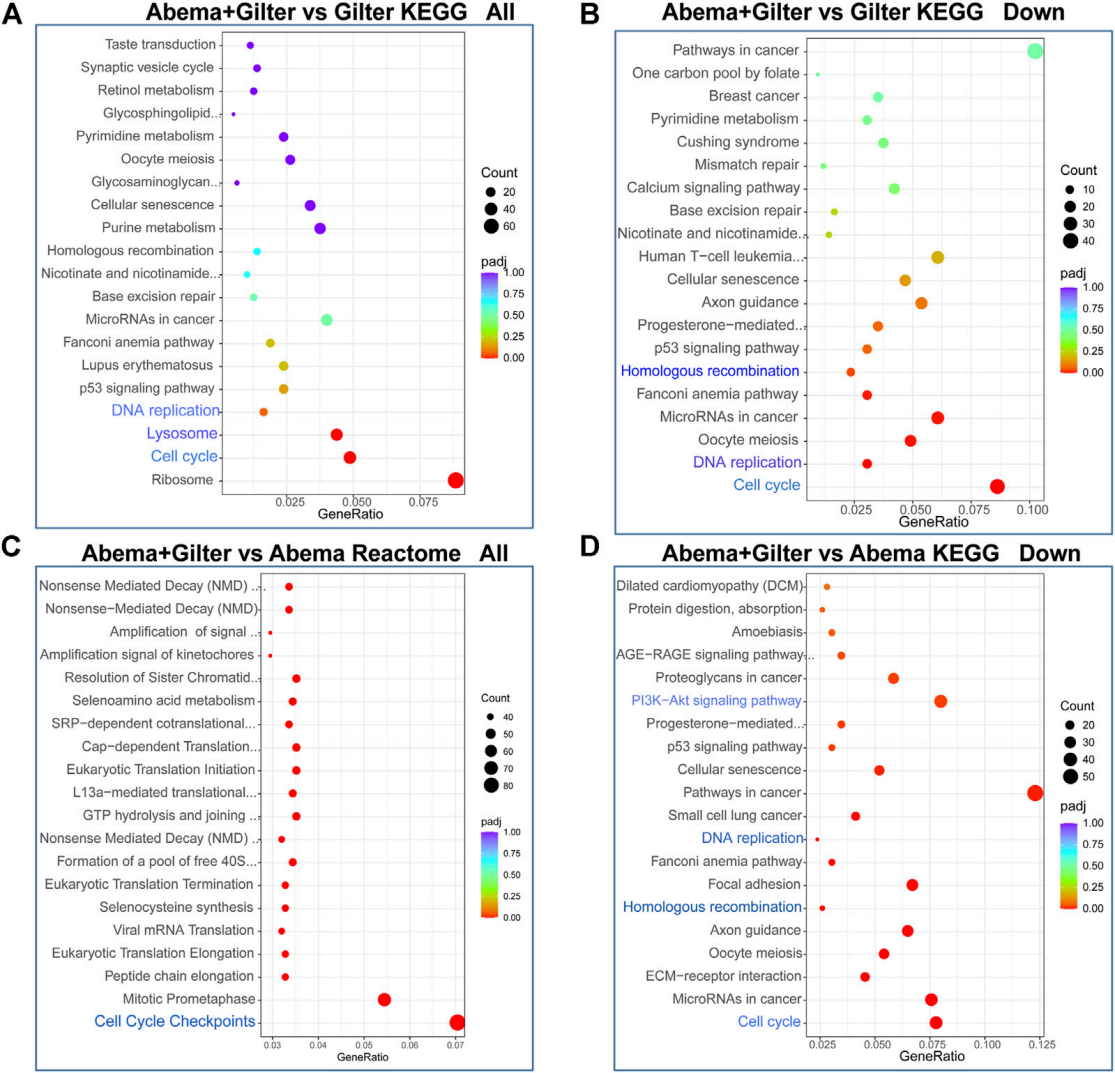
Abemaciclib in combination with gilteritinib affects cell cycle and homologous recombination pathways. A549 cells were treated by 1 μM abemaciclib, or 150 nM gilteritinib alone, or their combination for 48 h, the mRNA expressions were detected by RNA-seq. **(A–D)** Differentially expressed genes were subjected to Kyoto Encyclopedia of Genes and Genomes (KEGG) and Reactome.

To gain insight into the molecular mechanism of the synergistic anti-cancer activity of abemaciclib and gilteritinib, we performed transcriptome analysis of A549 cells by RNA-sequencing (RNA-seq) (Supplementary Figure S2A). Using KEGG and Reactome analysis of differentially expressed genes between different treatments, we found that cell cycle, DNA replication and homologous recombination (HR) pathways were among the most highly enriched pathways in the combination treatment-treated cells, when compared to gilteritinib alone treatment (Figures 4A,B). Consistently, cell cycle, DNA replication, HR, and AKT pathways were strikingly altered by gilteritinib in combination with abemaciclib treatment, compared to abemaciclib alone treatment (Figures 4C,D).

**FIGURE 5:**
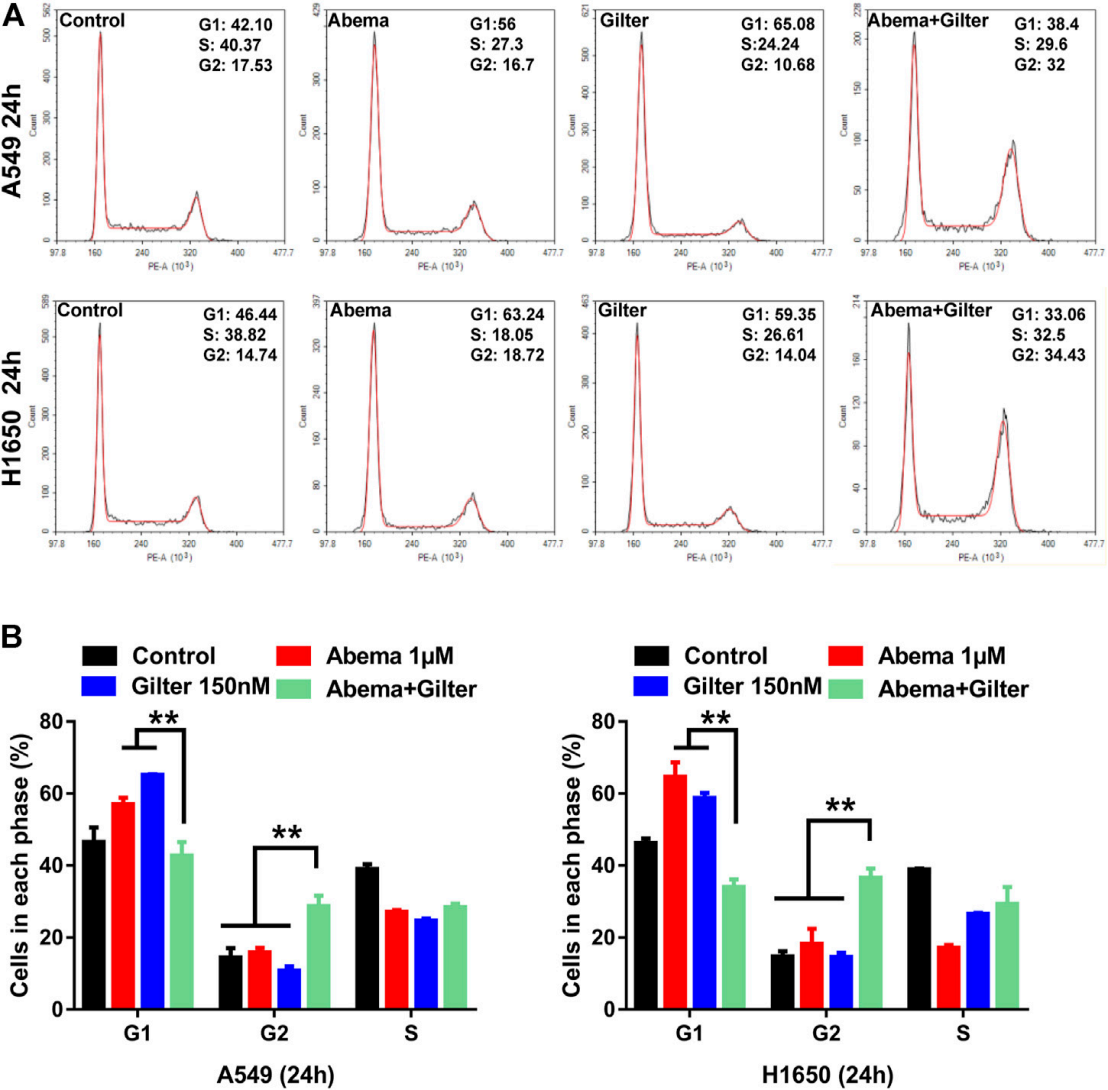
Abemaciclib in combination with gilteritinib induces cell-cycle arrest at G2 phase in lung cancer cells **(A,B)** A549 and H1650 cells were treated by 1 μM abemaciclib, or 150 nM gilteritinib alone, or their combination for 24 h, and cell cycle was determined by flow cytometry (*n* = 3). **p < 0.001.

To further validate the effect of combined treatment of gilteritinib and abemaciclib on cell cycle in lung cancer cells, we performed flow cytometry to detect cell cycle. Consistent with a previous report (Ma et al., 2020), abemaciclib treatment blocked G1/S cycle transition in A549, H1650 cells (Figures 5A,B). Consistent with abemaciclib, gilteritinib alone treatment resulted in a cell-cycle arrest at G1/M phase (Figures 5A,B). Interestingly, abemaciclib combined with gilteritinib promoted cell cycle progression at the G1/G2 phase transition and induced cell cycle arrest at the G2 phase (Figures 5A,B).

**FIGURE 6:**
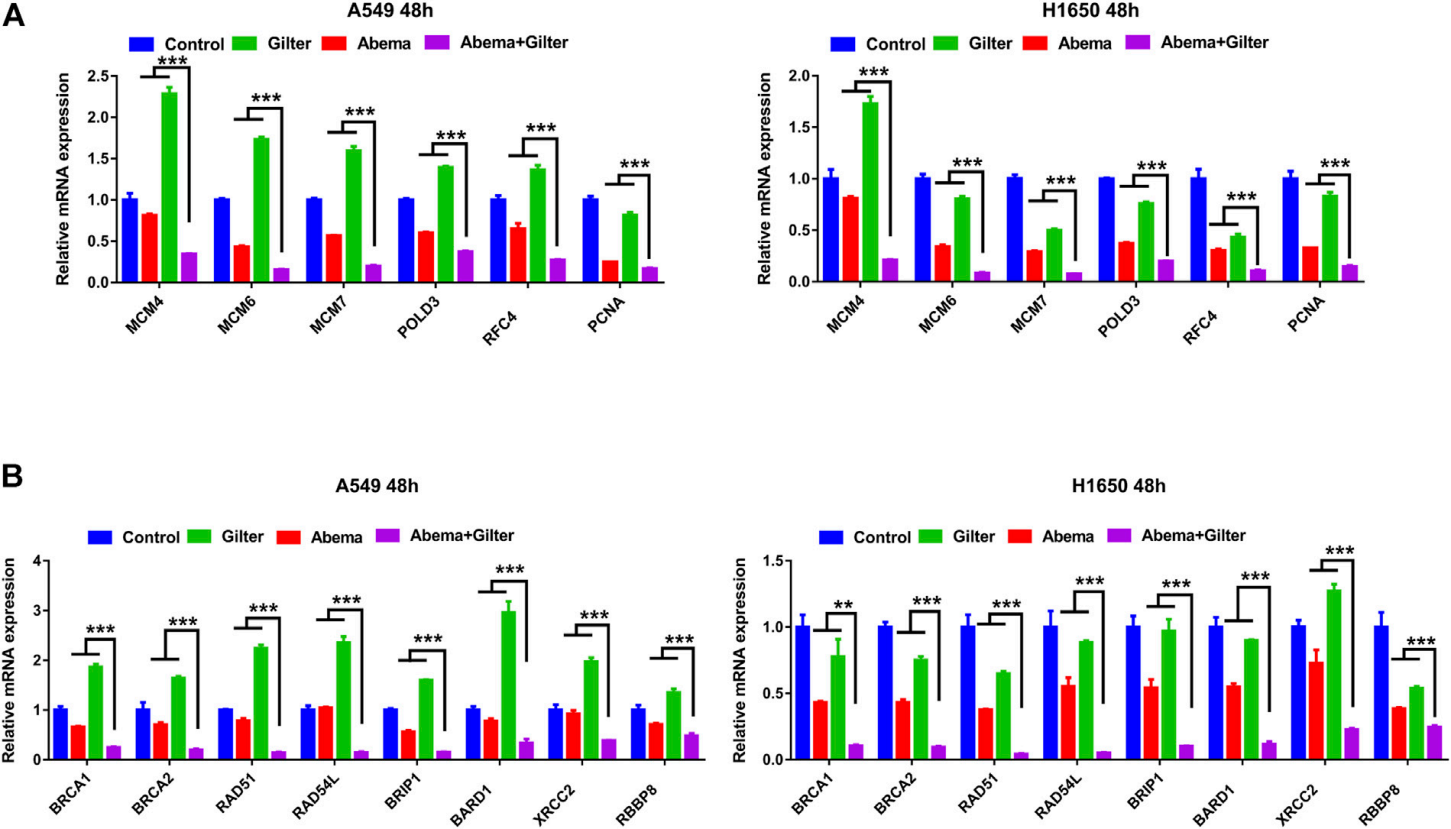
Abemaciclib in combination with gilteritinib inhibits DNA replication, HR associated gene expression **(A)** A549 and H1650 cells were treated by 1 μM abemaciclib alone, or 150 nM gilteritinib alone, or their combination for 48 h, and the expressions of DNA replication genes, including MCM4, MCM6, MCM7, POLD3, RFC4 and PCNA were determined by qRT-PCR **(B)** A549, H1650 cells were treated by 1 μM abemaciclib alone, or 150 nM gilteritinib alone, or their combination for 48 h, and the mRNA expressions of HR pathway gene, including BRCA1, BRCA2, RAD51, RAD54L, BRIP1, BARD1, XRCC2, RBBP8 were determined by qRT-PCR. Data are representative of three independent experiments. **p < 0.001. ***p < 0.0001.

We next performed RT-qPCR to ascertain whether the combined treatment affects DNA replication and homologous recombination (HR) pathway. As shown in Figure 6A, combined abemaciclib with gilteritinib significantly reduced the expressions of DNA replication genes, including MCM4, MCM6, MCM7, POLD3, RFC4 and PCNA in A549 and H1650 cells, compared to either drug. Consistent with this, the combined treatment effectively decreased the levels of HR pathway genes, including BRCA1, BRCA2, RAD51, RAD54L, BRIP1, BARD1, XRCC2 and RBBP8 in lung cancer cells, compared to either drug treatment (Figure 6B). Taken together, combination of abemaciclib with gilteritinib induced cycle arrest at G2 phase, and inhibited DNA replication and HR-associated gene expression.

### Combination of Abemaciclib and Gilteritinib Suppresses Lung Cancer Growth *In Vivo*

**FIGURE 7:**
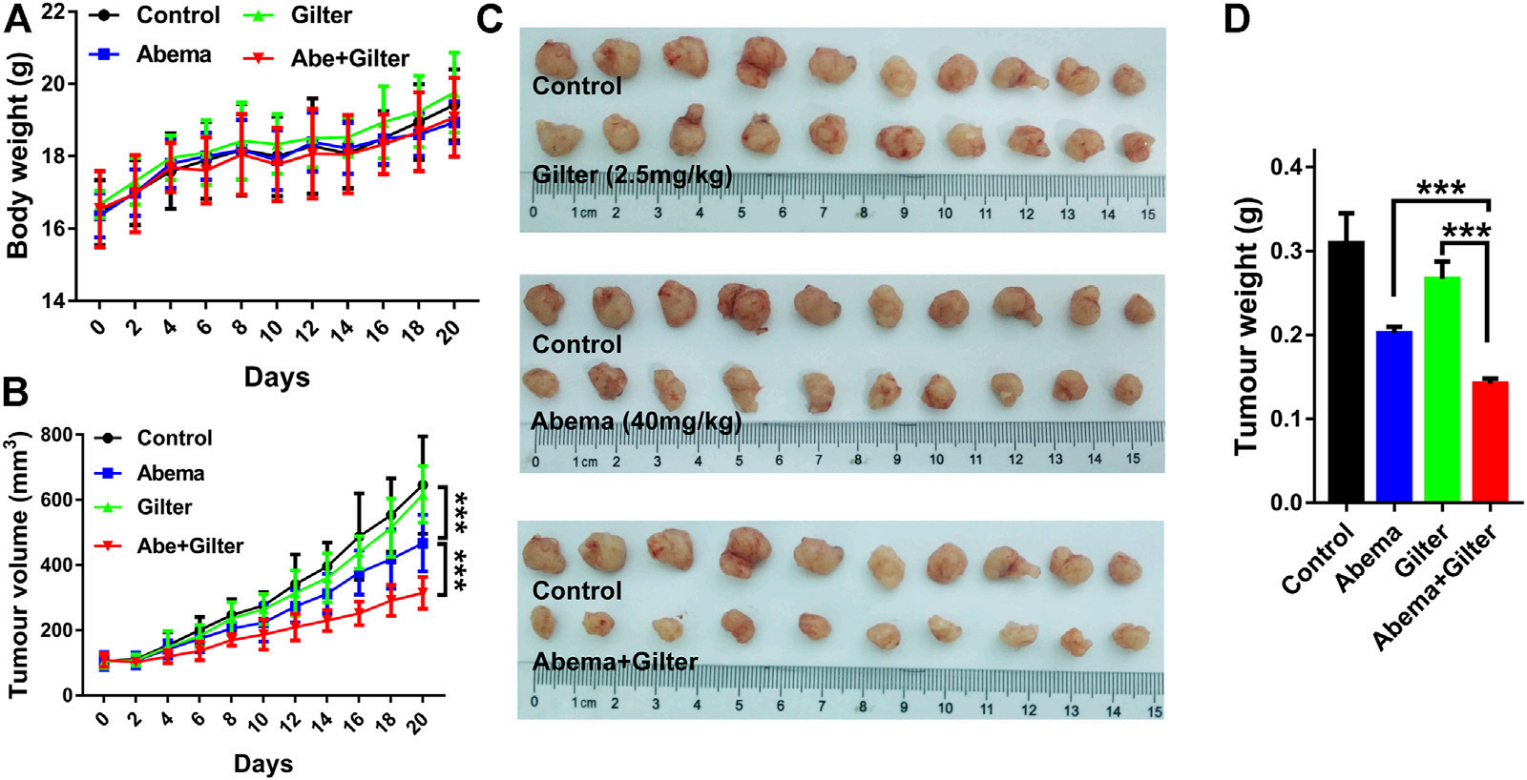
Abemaciclib in combination with gilteritinib suppresses lung cancer growth *in vivo*. A549 cells (4×10^6^) were subcutaneously injected into BALB/c nude mice. When tumors volume reached at 100 mm^3^, mice were randomly divided into four groups: control, abemaciclib (40 mg/kg), gilteritinib (2.5 mg/kg), abemaciclib plus gilteritinib (*n* = 10). Mice in treatment groups were administered daily *via* oral gavage, while mice in control group were treated with vehicle for continuous 20 days **(A,B)** Body weight and tumor volume were measured at every 2 days **(C)** After treatment, tumor tissues were collected, and the tumor inhibitory rate was calculated **(D)** The tumor weight was measured. ***p < 0.0001.

To validate whether gilteritinib sensitizes lung cancer cells to abemaciclib *in vivo*, we injected A549 cells into BALB/c nude mice to establish mouse xenograft model. Mice bearing A549 cells were respectively treated with the vehicle, abemaciclib, gilteritinib, or in combination for continuous 20 days. Treatment with abemaciclib, or gilteritinib alone, or their combination displayed no obvious toxicity in mice (Figure 7A). As shown in Figures 7B–D, abemaciclib alone treatment significantly suppressed the tumor growth (inhibitory rate, IR = 34.7%), while monotherapy with gilteritinib slightly inhibited the tumor growth (IR = 13%). Notably, their combination was more effective in inhibiting the tumor growth, compared with either abemaciclib or gilteritinib drug (IR = 54.6%). In addition, RNA-seq data revealed that the combination treatment was reasonable as a therapeutic strategy for lung cancer, and the side effects of their combination mainly included nervous and metabolic disorders (Supplementary Figures S2B,C). Overall, our data indicate that abemaciclib in combination with gilteritinib is an effective for the treatment of lung cancer *in vivo*.

## Discussion

CDK4/6 inhibitors are approved for the treatment of hormone receptor (HR)-positive, and human epidermal growth factor receptor 2 (HER2)-negative metastatic breast cancer (Spring et al., 2020). Mechanically, CDK4/6 inhibitors block cycle progression from G1 to S-phase by inactivating the retinoblastoma (Rb) protein (Lim et al., 2016). Considering that CDK4/6/Rb pathways are frequently upregulated in the lung cancer patients, CDK4/6 inhibitors are considered as a safe and effective strategy for treating lung cancer (Lim et al., 2016; Patnaik et al., 2016). However, clinical trials show that the use of CDK4/6 inhibitors, including abemaciclib, are limited for treatment of lung cancer duo to its poor efficacy (Pacheco and Schenk, 2019). Thus, drug combination is a promising approach to improve the anti-cancer effect of CKD4/6 inhibitors. Here, we combined several FDA-approved small-molecule protein kinase inhibitors with abemaciclib to co-treat lung cancer cells, and identified gilteritinib as a candidate to sensitize abemaciclib. The combination treatment suppressed cell proliferation, and induced senescence, apoptosis, cell cycle arrest, and massive vacuoles formation in lung cancer cells. Mechanistically, the combination treatment inhibited Rb and AKT, DNA replication, and HR-associated gene expressions.

Cellular senescence is a defensive mechanism for host in response to unnecessary damage, and senescent cells are accompanied by a failure to re-enter the transition of cell cycle (Calcinotto et al., 2019; Lee and Schmitt, 2019). Abundant evidences have demonstrated that induction of senescence is an excellent strategy for the treatment of cancer (Nardella et al., 2011; Wang et al., 2019; Ruscetti et al., 2020). Rb is a critical senescence-associated gene, and senescence is an important therapeutic response to CDK4/6 inhibitors (Aliagas et al., 2021; Maskey et al., 2021). Here, we demonstrated that combined abemaciclib with gilteritinib treatment triggered more senescence than either drug, in lung cancer cells. In addition to cell senescence, interestingly, the combination treatment was able to induce cytoplasmic vacuolization in lung cancer cells. Excessive vacuoles can trigger cytoplasmic membrane rapture, and metabolic disorder, eventually leading to cell death, termed “methuosis” (Overmeyer et al., 2011). Several small-molecule compounds, including abemaciclib, are inducers of methuosis, which display dramatic anti-tumor activities (Robinson et al., 2012; Huang et al., 2018; Hino et al., 2020). Interestingly, we found that gilteritinib treatment alone not only induced large amounts of vacuoles, but also enhanced abemaciclib-induced vacuolization in lung cancer cells. Notably, abemaciclib is a unique CDK4/6 inhibitor different from ribociclib and palbociclib (Chong et al., 2020), where only abemaciclib alone treatment or in combination with gilteritinib triggered vacuolization. As a result, abemaciclib, rather than other CDK4/6 inhibitors (ribociclib, palbociclib), synergized with gilteritinib to inhibit the growth of lung cancer cells. In addition, quizartinib is an another selective FLT3 (target of gilteritinib) inhibitor that is also effective to combine with abemaciclib to inhibit proliferation of lung cancer cells, suggesting that FLT3 might confer synthetic lethality to CDK4/6 inhibition. Thus, combined abemaciclib with gilteritinib is a distinct and reasonable drug combination for the treatment of lung cancer.

The tumor suppressor, Rb interacts with E2F transcription factor to regulate cell cycle, and Rb is initially phosphorylated by CDK4/6 signaling (Dick et al., 2018). Thus, CDK4/6 inhibitors, including abemaciclib, inhibit Rb-E2F pathways to induce cell cycle arrest at G1-phase. Correspondingly, we demonstrated that abemaciclib or gilteritinib alone induced G1-phase cycle arrest, but combination of abemaciclib with gilteritinib induced cell cycle arrest at G2-phase. However, these results are not very consistent with Western blot data that combined treatment completely inhibited Rb phosphorylation, and the underlying mechanism remains unclear. Considering that the AKT pathway is identified as an important target of gilteritinib (Li et al., 2020), we hypothesized that the combination treatment affects AKT pathway. To our surprise, reactivation of AKT pathway was observed in gilteritinib-treated cells, however, such reactivation was almost completely blocked by the addition of abemaciclib treatment. Importantly, combined abemaciclib and gilteritinib treatment was able to inhibit DNA replication and HR-associated genes expression. Thus, these results robustly provide a rationale for combination of abemaciclib with gilteritinib to treat lung cancer.

In conclusion, our data supported the combination of abemaciclib and gilteritinib as an excellent therapeutic candidate for treatment of lung cancer, whereby the combined treatment displays greater cytotoxicity by inhibiting Rb, AKT, DNA replication and HR-associated genes expression than either drug. In addition, mouse xenograft model showed that the combination of abemaciclib and gilteritinib treatment is effective and much tolerated in mice. Regardless, further clinical investigations are warranted to evaluate the therapeutic efficacy of the combined treatment.

## Data Availability

The original contributions presented in the study are included in the article/Supplementary Material, further inquiries can be directed to the corresponding author.

## Supplementary Material

Supplementary Figure S1
**(A)** HCT115, KYSE30 cells were treated by abemaciclib, or gilteritinib alone, or their combination for 72 h, and the proliferation was measured using crystal violet staining. **(B)** H1650 cells treated by palbociclib, or gilteritinib alone, or their combination for 72 h, and the proliferation was measured using crystal violet staining. **(C)** H1650 cells treated by abemaciclib, or quizartinib alone, or their combination for 72 h, and the proliferation was measured using crystal violet staining. **(D)** HCT115 cells were treated by 1 μM abemaciclib, or 150 nM gilteritinib alone, or their combination for 72 h, and cellular morphology was observed using microscope. **(E)** A549 cells were treated by 25 μM palbociclib, or 150 nM gilteritinib alone, or their combination for 72 h, and cellular morphology was observed using microscope. **(F)** Abemaciclib-resistant A549 cells were developed with increasing dose-escalation of abemaciclib for 3 months drug exposure. Parental or abemaciclib -resistant A549 cells were treated by abemaciclib or gilteritinib, and the proliferation was measured using crystal violet staining. Data are representative of three independent experiments.

Supplementary Figure S2
**(A)** A549 cells were treated by 1 μM abemaciclib, or 150 nM gilteritinib alone, or their combination for 48 h, the mRNA expressions were detected by RNA-seq. **(B, C)** Differentially expressed genes were subjected to disease ontology (DO) analysis.

Supplementary Figure S3
**(A)** A549, H1650 cells were treated by 1 μM abemaciclib, or 150 nM gilteritinib alone, or their combination for 48 h, and Western blot was used to determine the expressions of LC3, p-Rb (ser807/811) and Rb. **(B)** A549 cells were treated by 1 μM abemaciclib, or 150 nM gilteritinib alone, or their combination for 24 h, and Western blot was used to determine the expressions of p-Rb (ser807/811, ser780). **(C)** A549 cells were treated by 1 μM abemaciclib, or 50 nM rapamycin alone, or 15 μM Torin1, or their combination for 48 h, and cellular morphology was observed using microscope.
