# Supplementary figures and images for: Gilteritinib Enhances Anti-Tumor Efficacy of CDK4/6 Inhibitor, Abemaciclib in Lung Cancer Cells
Source: Front Pharmacol. 2022 Jun 23;13:829759. doi: 10.3389/fphar.2022.829759 (PMC9262324; doi:10.3389/fphar.2022.829759)

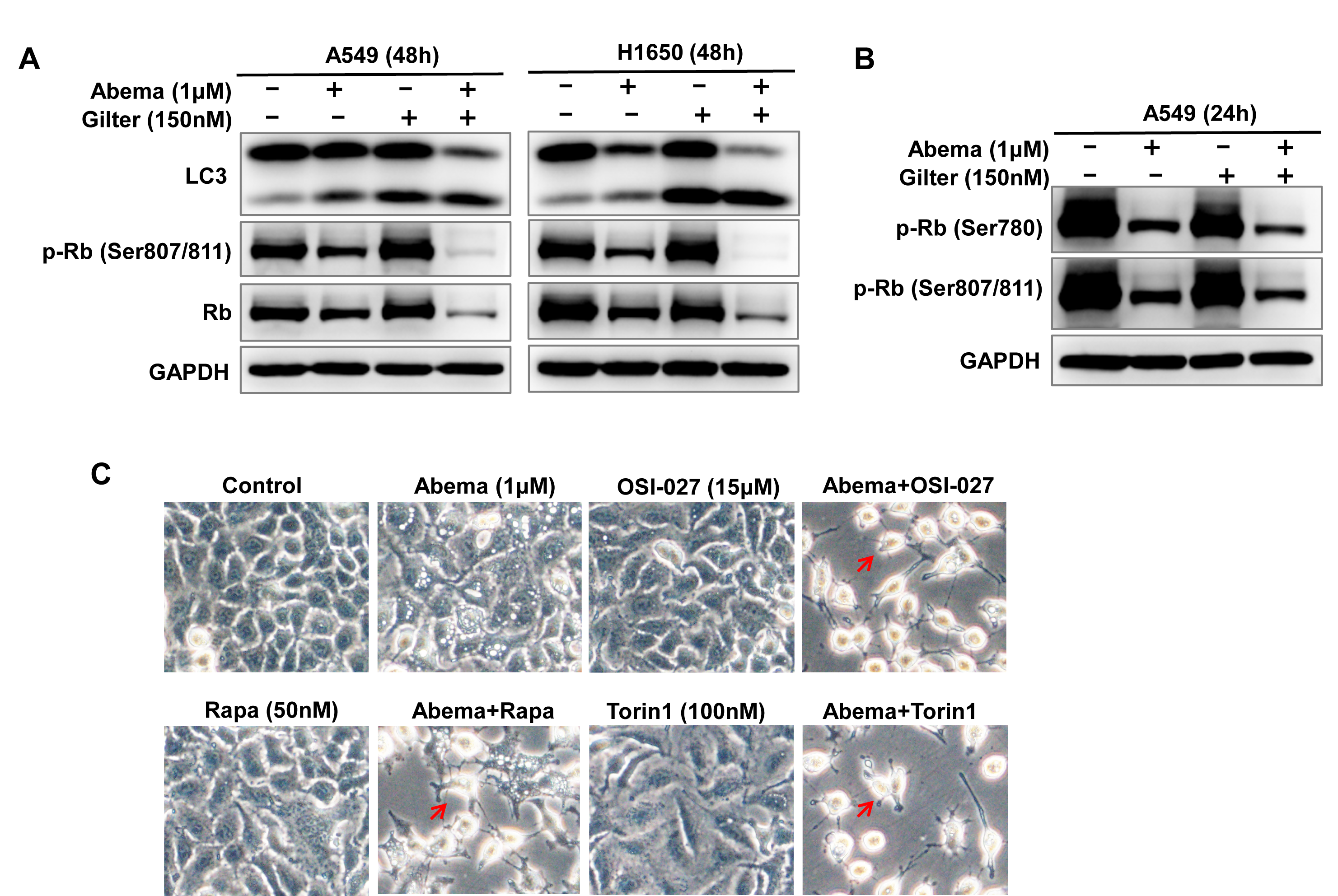

Supplement: Supplementary file 1 [file Image3.tif]

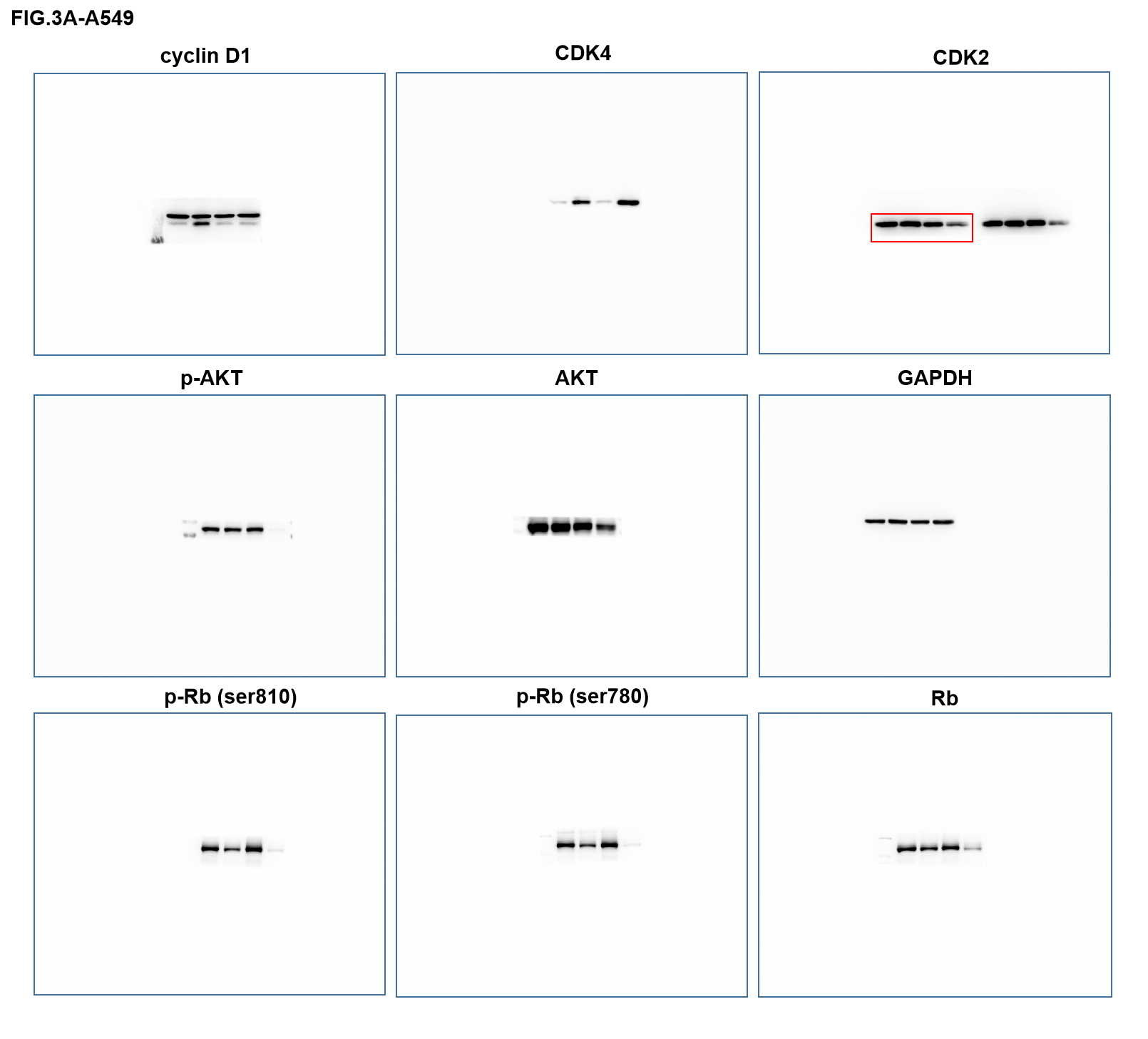

Supplement: Supplementary file 2 [file DataSheet1.ZIP › Western blots/Supplementary FIG.3.tif]

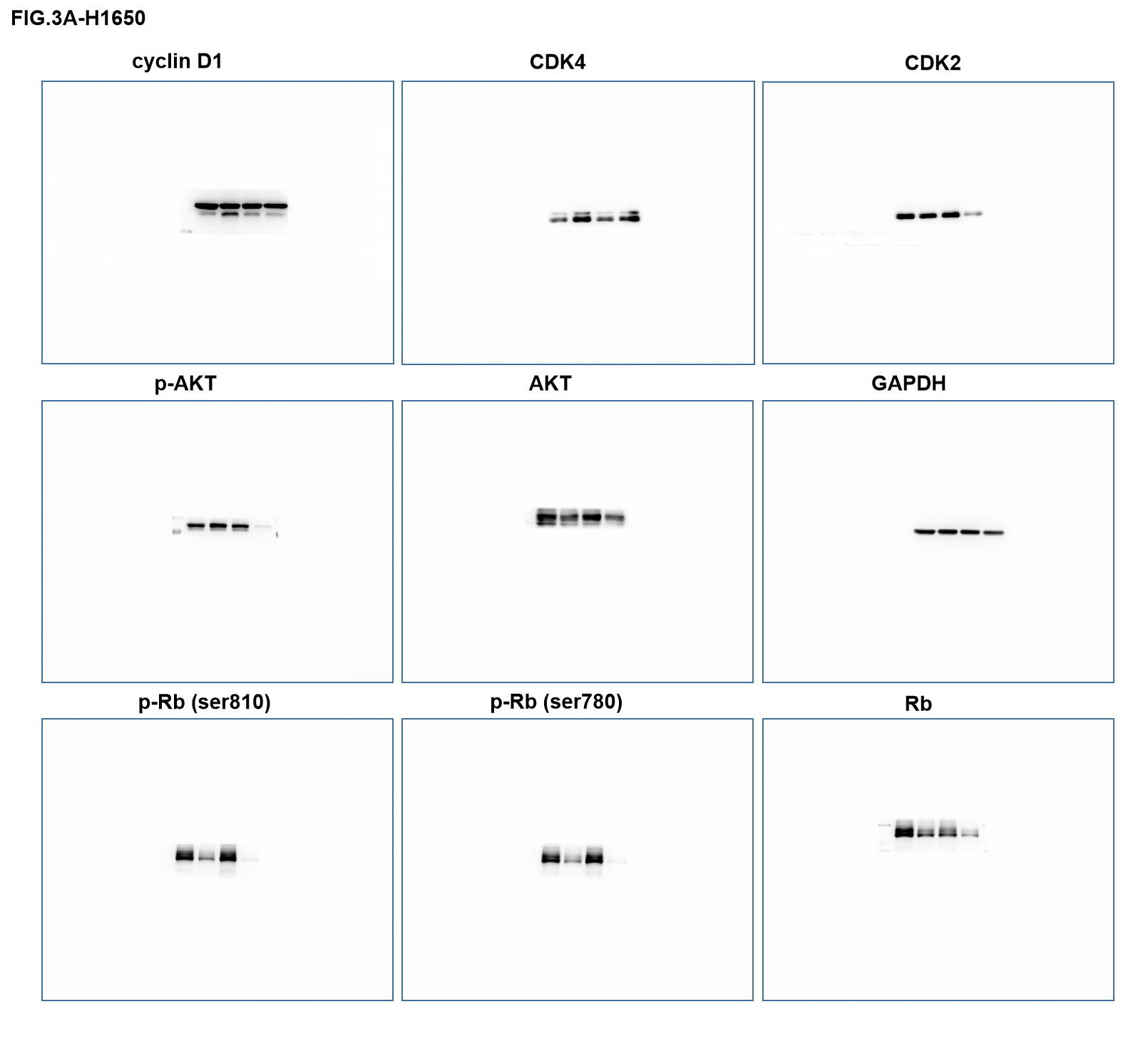

Supplement: Supplementary file 2 [file DataSheet1.ZIP › Western blots/Supplementary FIG.4.tif]

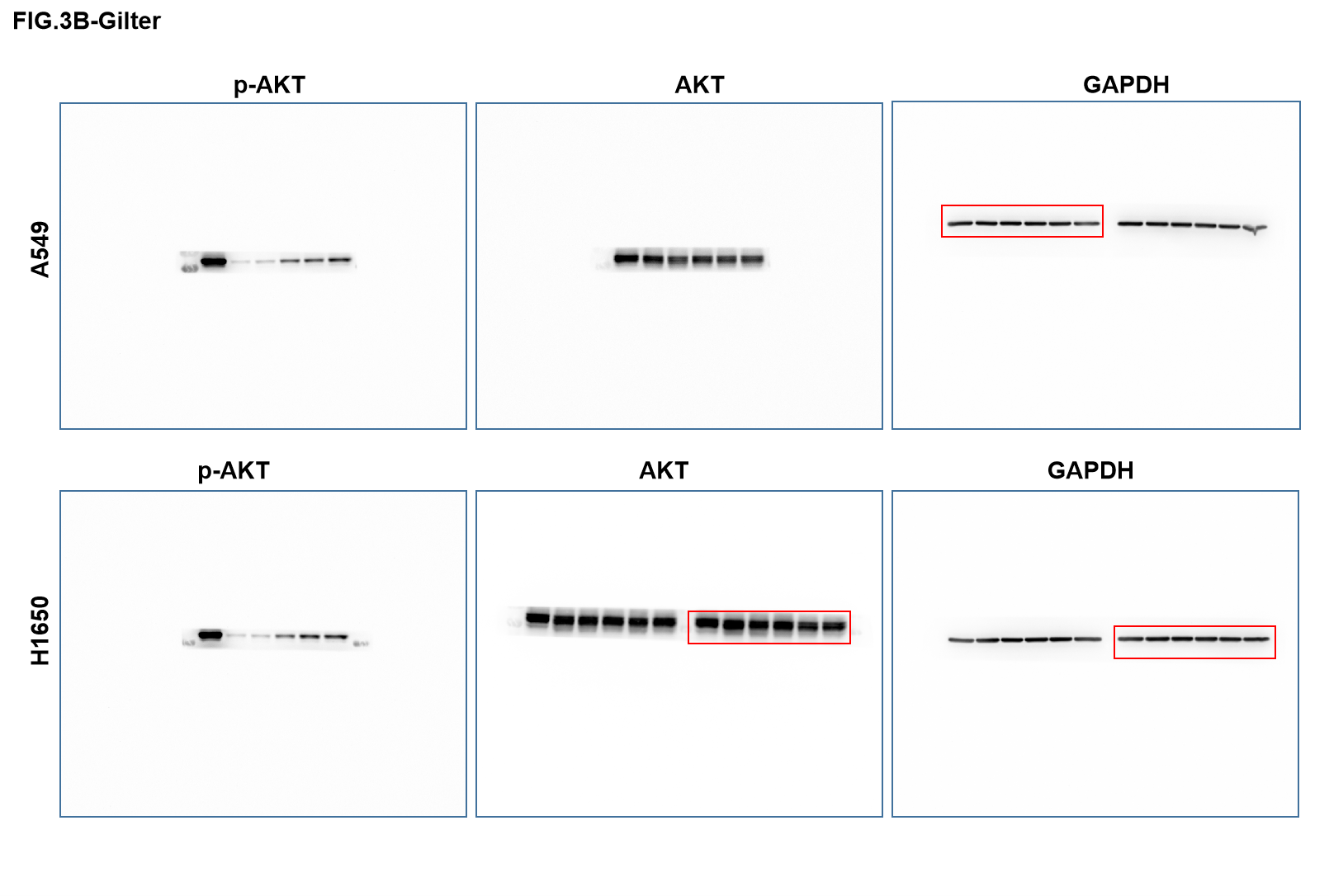

Supplement: Supplementary file 2 [file DataSheet1.ZIP › Western blots/Supplementary FIG.5.tif]

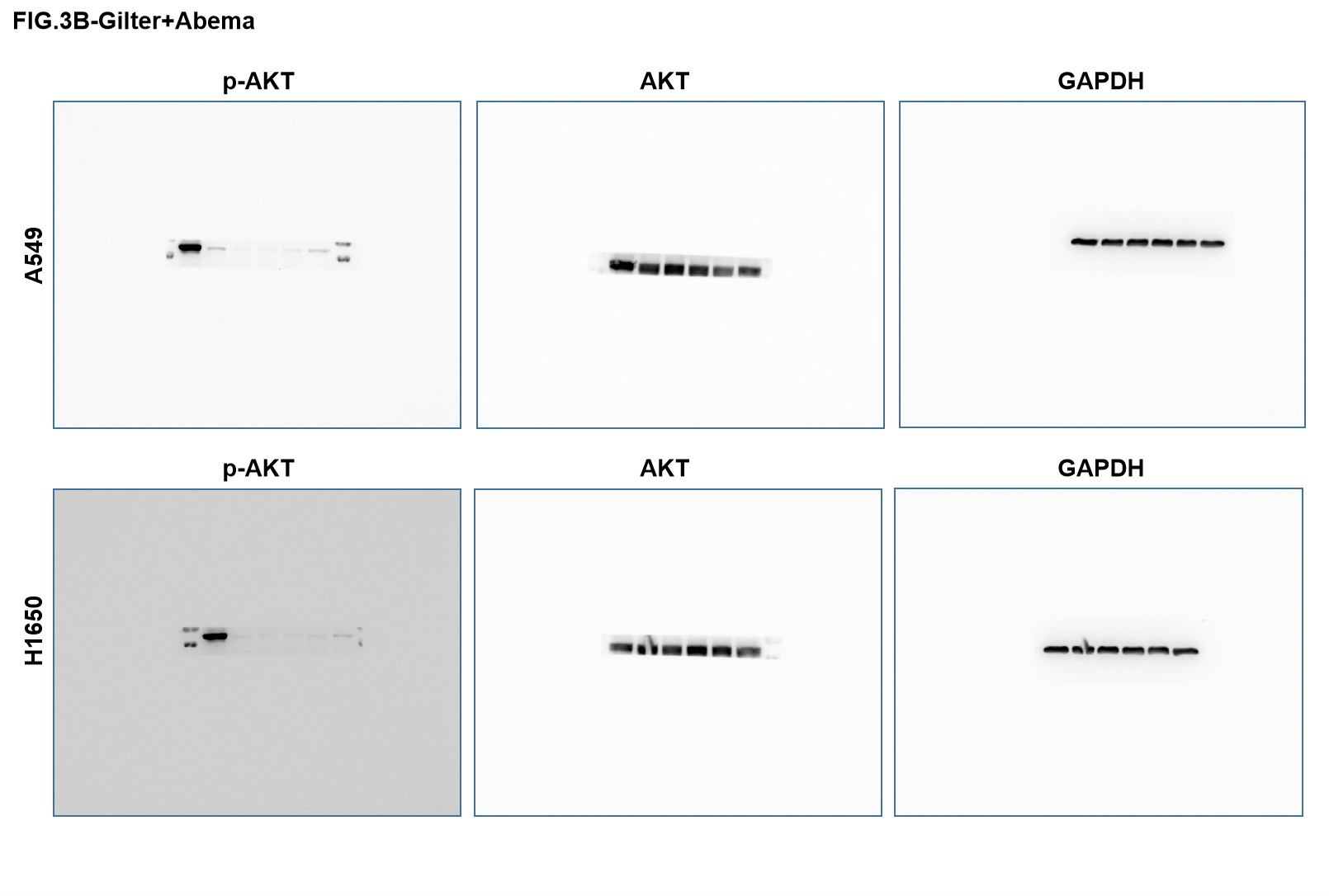

Supplement: Supplementary file 2 [file DataSheet1.ZIP › Western blots/Supplementary FIG.6.tif]

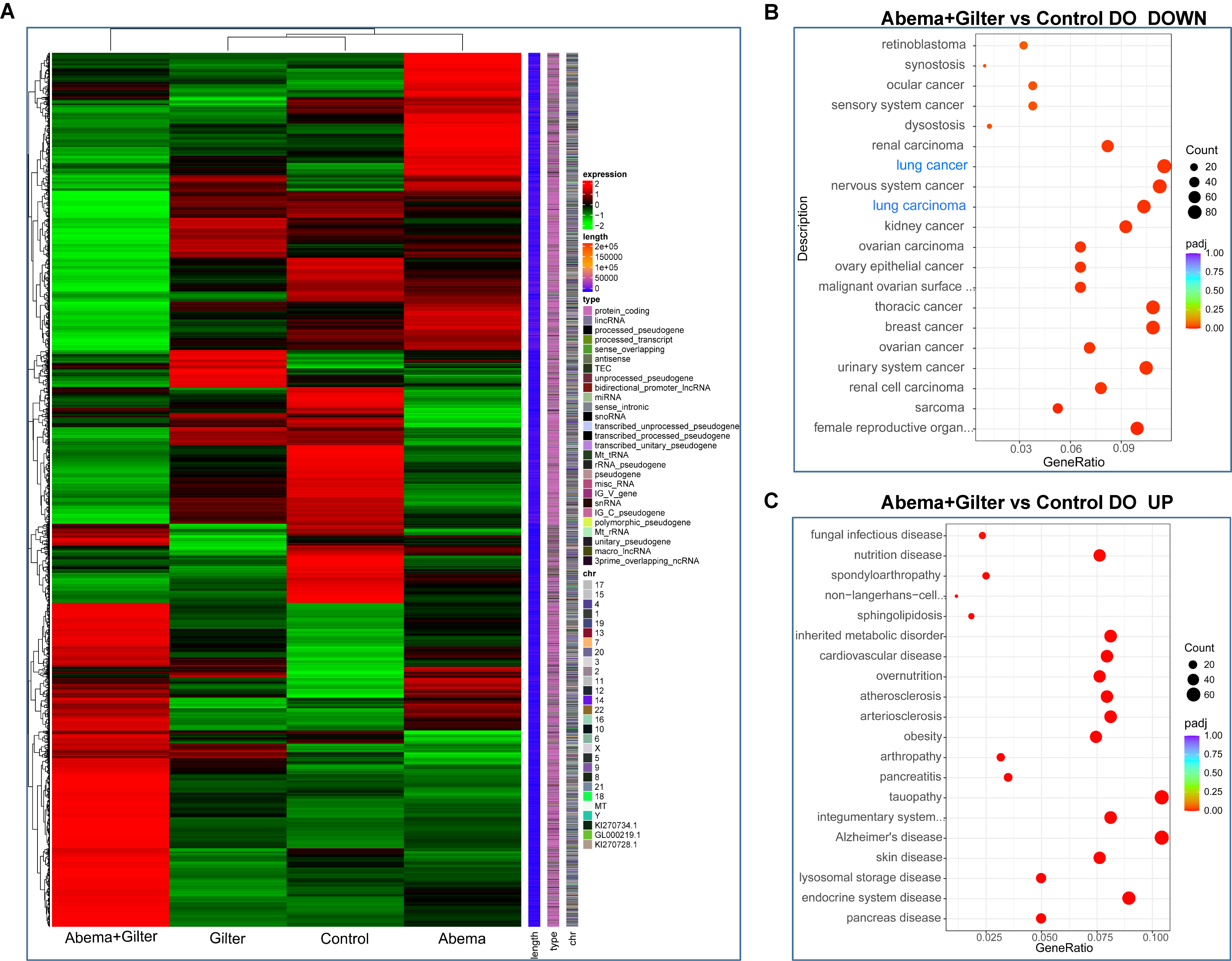

Supplement: Supplementary file 3 [file Image2.tif]

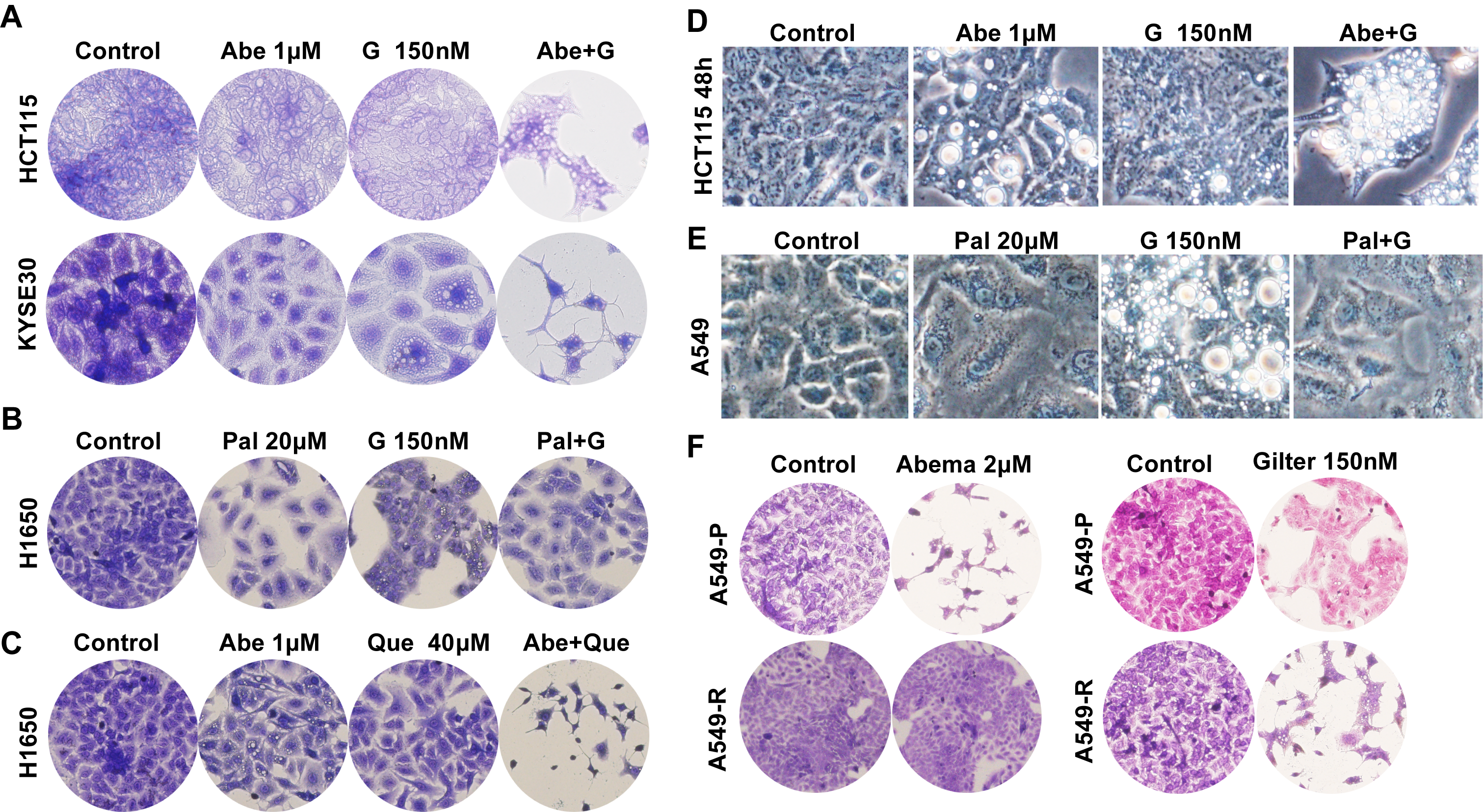

Supplement: Supplementary file 4 [file Image1.tif]
